# Geminivirus Resistance: A Minireview

**DOI:** 10.3389/fpls.2020.01131

**Published:** 2020-07-23

**Authors:** Kayla Beam, José Trinidad Ascencio-Ibáñez

**Affiliations:** Department of Molecular and Structural Biology, North Carolina State University, Raleigh, NC, United States

**Keywords:** geminivirus, resistance, genetic resistance, agriculture, genetic engineering

## Abstract

A continuing challenge to crop production worldwide is the spectrum of diseases caused by geminiviruses, a large family of small circular single-stranded DNA viruses. These viruses are quite diverse, some containing mono- or bi-partite genomes, and infecting a multitude of monocot and dicot plants. There are currently many efforts directed at controlling these diseases. While some of the methods include controlling the insect vector using pesticides or genetic insect resistance (Rodríguez-López et al., 2011), this review will focus on the generation of plants that are resistant to geminiviruses themselves. Genetic resistance was traditionally found by surveying the wild relatives of modern crops for resistance loci; this method is still widely used and successful. However, the quick rate of virus evolution demands a rapid turnover of resistance genes. With better information about virus-host interactions, scientists are now able to target early stages of geminivirus infection in the host, preventing symptom development and viral DNA accumulation.

## Introduction: Viral Proteins May Be Targets for Resistance

Geminiviruses are circular single-stranded DNA viruses that infect a wide range of plant species including many important crops. Damages attributed to geminiviruses include over $300 million in loss in the Indian bean industry (Patil et al., 2014), up to 100% loss of tomato crop in Italy and the Dominican Republic (Picó et al., 1996), and nearly $2 billion loss in African cassava production (Patil and Fauquet, 2009). The impact of geminiviruses is widespread and destructive. The family Geminiviridae has nine genera based on viral genome structure and insect vectors. In the case of begomoviruses, genomes can be mono- or bipartite, with each circular DNA (~2.5 Kb) packaged in a twinned icosahedral particle (Zerbini et al., 2017).

**Figure 1:**
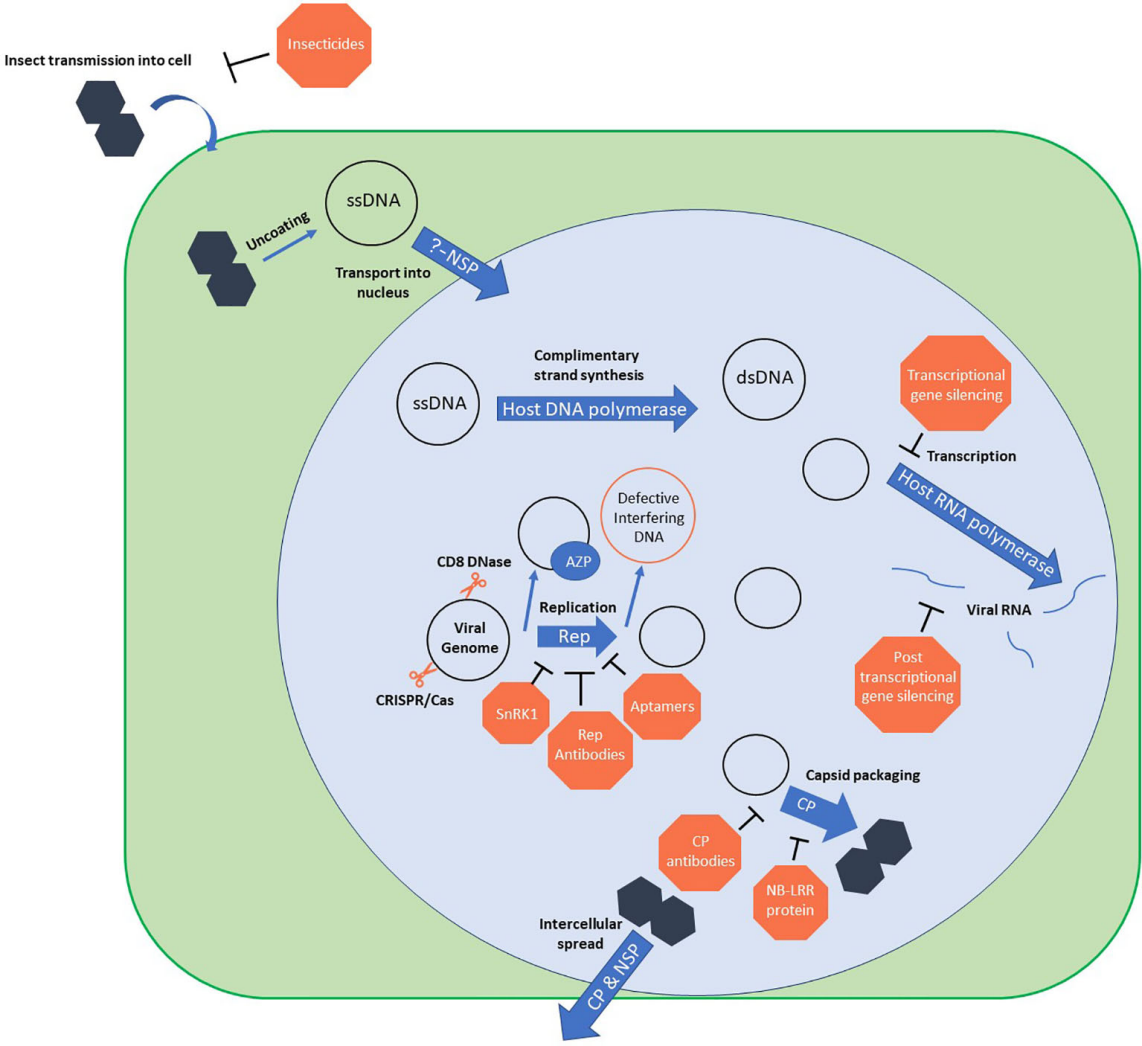
Geminivirus life cycle with points of resistance. Orange blocks show points where known resistance mechanisms interfere with the virus. ssDNA, single stranded DNA; dsDNA, double stranded DNA; NSP, nuclear shuttle protein; AZP, artificial zinc-finger protein; Rep, replication-associated protein; CP, coat protein.

Geminivirus infection begins when an insect vector containing virions feeds on a host plant. The viral genome is deposited and unpackaged in the phloem cells. A complementary strand is synthesized, then the dsDNA is replicated and packaged into mini-chromosomes using host histones (reviewed in Jeske, 2009 and Hanley-Bowdoin et al., 2013). The viral genes are transcribed by host RNA Polymerase II. The viral genome is replicated by rolling-circle and recombination-dependent replication systems (Jeske et al., 2001). These processes require both host and viral proteins. See **Figure 1** for an overview of the geminivirus life cycle.

The most critical geminiviral protein for virus replication is the replication initiator protein (Rep). It initiates replication by binding the origin sequence, nicking the DNA, and associating with host factors (Laufs et al., 1995; Kong et al., 2000; Arguello-Astorga et al., 2004; Desvoyes et al., 2006). The geminiviral transcriptional activator protein (TrAP) is involved in pathogenicity and suppresses both transcriptional and post transcriptional gene silencing (Sunter and Bisaro, 1992; Hong et al., 1996; Voinnet et al., 1999; Shivaprasad et al., 2005; Trinks et al., 2005; Wang et al., 2005; Chowda-Reddy et al., 2009; Castillo-González et al., 2015; Kumar et al., 2015). Furthermore, the replication enhancer protein (REn) contributes to increased viral replication and interacts with host factors and with Rep. Geminiviruses also encode proteins for packaging and movement. The coat protein (CP) comprises the viral capsid and is critical for vector specificity (Noris et al., 1998) and viral nuclear import (Liu et al., 1999) and is also required for symptom development. In bipartite geminiviruses, the nuclear shuttle protein (NSP) is responsible for nuclear import by binding ssDNA. It also aids in intercellular movement by interacting with the movement protein (MP) at the membrane. MP is responsible primarily for the intercellular spread of viral DNA (Noueiry et al., 1994) by increasing the size exclusion limit of plasmodesmata (Rojas et al., 2001). For excellent reviews in these subjects please see Hanley-Bowdoin et al., 2013; Czosnek et al., 2017; Rojas et al., 2018, and Martins et al., 2020.

## Naturally Occurring Resistance

In the process of crop domestication, traits that are not directly beneficial are often bred out in favor of those increasing yield in particular conditions. When a new challenge arises, breeders search wild relatives for traits that increase survival. This is the case with geminiviruses: there are multiple geminivirus resistance genes from undomesticated relatives that are utilized in agriculture. Examples include many crops like beans (reviewed by Blair and Morales, 2008) or cotton (Zaidi et al., 2020). This minireview will cover resistance genes in tomato and cassava in detail. A selected list of resistance genes for these crops is presented in **Table 1**.

**Table 1:** List of genes involved in geminivirus resistance and geminiviral suppressors of silencing.

Gene	Protein	Plant	Virus	Reference
*Ty-1, Ty-3 *	RNA-dependent polymerase	*S. lycopersicum*	TYLCV	(Zamir et al., 1994; Verlaan et al., 2013; Butterbach et al., 2014)
*Ty-2 *	NB-LRR class protein	*S. lycopersicum*	TYLCV	(Yang et al., 2014; Yamaguchi et al., 2018)
*Ty-4 *		*S. lycopersicum*	TYLCV	(Ji et al., 2009)
*ty-5 *	pelota	*S. lycopersicum*	TYLCV	(Hutton et al., 2012; Scott et al., 2015; Lapidot et al., 2015)
*Ty-6 *		*S. lycopersicum*	TYLCV	(Scott et al., 2015)
*CMD1, CMD3 *		*M. esculenta*	ACMV	(Fregene et al., 2000; Akano et al., 2002; Okogbenin et al., 2012)
*CMD2 *		*M. esculenta*	ACMV	(Akano et al., 2002; Kuria et al., 2017)
*CchGLP *	MN-SOD	*C. chinense*	PHYVV, PepGMV	(León-Galván et al., 2011; Guevara-Olvera et al., 2012; Mejía-Teniente et al., 2015)
*SlSnRK1 *	SnRK1	*S. lycopersicum*	TYLCV, CaLCuV	(Shen et al., 2011; Shen et al., 2014; Hulsmans et al., 2016)
*Permease-1 like *		*S. habrochaites* introgressed *S. lycopersicum*	TYLCV	(Eybishtz et al., 2009)
*LeHT1 *	hexose transporter	*S. lycopersicum*	TYLCV	(Eybishtz et al., 2010; Sade et al., 2012)
*SlVSRLip *	lipocalin-like	*S. lycopersicum*	TYLCV	(Sade et al., 2012)
*NIK *	NSP-interacting kinase	*A. thaliana, S. lycopersicum, G. max*	begomoviruses	(Brustolini et al., 2015)
*SGS3 *	RNA binding protein in PTGS	*N. benthamiana*	TYLCV	(Li et al., 2017)
*WRKY *	Group III transcription factors	*S. lycopersicum*	TYLCV	(Huang et al., 2016)

*Solanum chilense* is the most popular wild tomato relative for introgressing Tomato yellow leaf curl virus (TYLCV) resistance, as over 80% of its accessions are resistant (Yan et al., 2018). Many of the *Ty* family of resistance loci are from this plant. *Ty-1* was introgressed into tomato from *S. chilense* and mapped to chromosome 6. It confers a tolerant symptomless response against TYLCV in homozygous plants (Zamir et al., 1994). *Ty-1* is allelic with another resistance gene from *S. chilense*, *Ty-3*. These genes encode an RNA-dependent RNA polymerase (RDR) similar to RDRs 3, 4, and 5 in *Arabidopsis thaliana*, implying a role for RNA interference (Verlaan et al., 2013). *A. thaliana* itself may be a source for resistance as found by Reyes et al. (2017) in an accession showing immunity to Cabbage leaf curl virus (CbLCV) and Beet curly top virus (BCTV). *Ty-1* plants have increased siRNA levels in comparison to non-resistant cultivars, corresponding with high levels of viral DNA methylation. This indicates transcriptional gene silencing (TGS) is likely involved in *Ty-1/Ty-3* mediated resistance (Butterbach et al., 2014).

*Ty-2* is a TYLCV resistance locus on tomato chromosome 11 introgressed from *Solanum habrochaites* (Yang et al., 2014). This locus hosts a gene known as *TYNBS1* that encodes an Nucleotide Binding, Leucine Rich Repeat (NB-LRR) protein, known elsewhere to provide pathogen resistance (Yamaguchi et al., 2018). The recessive *ty-5* locus from a hybrid tomato known as Tyking has been mapped to chromosome 4. It is closely tied to the Quantitative trait loci (QTL) marker *SlNAC1* (Anbinder et al., 2009; Hutton et al., 2012). The gene responsible for *ty-5* resistance is *pelota*, which encodes an mRNA surveillance factor homolog (Lapidot et al., 2015; Wang Y. et al., 2018). *Ty-4* and *Ty-6* are other resistances genes from *S. chilense* that are less understood. *Ty-4* is a minor locus on tomato chromosome 3 (Ji et al., 2009). *Ty-6* lies on chromosome 10, has incomplete dominance, and protects tomatoes against Tomato mottle virus (ToMoV) and TYLCV (Scott et al., 2015; Gill et al., 2019).

Cassava mosaic disease (CMD) is caused by multiple cassava mosaic geminiviruses, often in complexes. *CMD* has only three markers in cassava known to confer resistance. *CMD2* is a single locus preferred by breeders due to its dominance (Akano et al., 2002). However, *CMD2* is monogenic and thus at risk of viral evolution overcoming resistance. Monogenic resistance is not sufficient for long-term disease resistance, requiring constant innovation to keep ahead of viral evolution. The newest marker associated with CMD resistance is *CMD3* (Okogbenin et al., 2012). It arose through crossbreeding of cultivars with the *CMD2* locus and another recessive resistance locus, *CMD1*, and appears to provide the highest resistance level of the three (Kuria et al., 2017).

The *CMD2*-provided resistance is lost when the plants are regenerated through somatic embryogenesis (Beyene et al., 2016). The mechanism for this change is unknown. The other CMD resistance alleles (*CMD1* and *CMD3*) are unaffected and still reliable for use. The loss of *CMD2*-based resistance could provide a method to predict if other traits will share the same phenomenon (Chauhan et al., 2018). There are several potential mechanisms for this effect including somaclonal variation, which is caused by epigenetic changes when the cell undergoes the disorganized regeneration phase in tissue culture (Bairu et al., 2011; Lee and Seo, 2018). However, virus evolution through pseudorecombination or recombination and not changes in the host can also be at play to break resistance in the field.

This ability to overcome genetic resistance is a very challenging aspect in geminivirus-plant interactions. One such example is the discovery of sequences enhancing geminivirus symptoms (SEGS) during CMD infection. These SEGS (encoded in the cassava genome) enabled the virus to cause symptoms in otherwise resistant cultivars, breaking the resistance (Ndunguru et al., 2016). Viruses themselves also have a high rate of mutation that can change the virus-host interactions to evade resistance. For TYLCV, the mean rate of genomic substitutions is estimated to be 2.88 x 10^-4^ nucleotide substitutions per site every year (Duffy and Holmes, 2008). This ability of geminiviruses to overcome genetic resistance has led to a resurgence of Cotton leaf curl disease (CLCuD) in south Asia after it had been nearly eradicated (Amrao et al., 2010).

## Geminivirus-Plant Interactions and Understanding Host Resistance

Reverse genetics is a common method for identifying genes involved in viral infection. Eybishtz et al. used this method in 2009 to identify Permease I-like protein as a resistance factor against TYLCV. In knockout resistant plants, viral genomic titer increased and resulted in susceptibility (Eybishtz et al., 2009). Similarly, the hexose transporter *LeHT1* was also identified as a resistance gene. In *LeHT1*-knockout plants, TYLCV accumulates and causes a necrotic response. This implies programmed cell death may be a factor in geminiviral response (Eybishtz et al., 2010). A downstream gene in the same *LeHT1* pathway, a lipocalin-like gene, also results in a necrotic response to TYLCV when mutated from the resistant tomato (Sade et al., 2012).

A separate geminivirus resistance gene that also involves programmed cell death includes the germin-like *CchGLP* gene found in resistant peppers. This protein has manganese superoxide reductase activity which increases upon infection by Pepper golden mosaic virus or Pepper huasteco yellow vein virus in resistant plants only (León-Galván et al., 2011). Resistant peppers with knocked-out *CchGLP* develop symptoms, indicating this gene has an important role in defense (Mejía-Teniente et al., 2015). When transferred into susceptible tobacco plants, *CchGLP* conferred a mild symptom phenotype with increased reactive oxygen levels and expression of systemic acquired resistance-related genes (Guevara-Olvera et al., 2012). Reactive oxygen species promote programmed cell death, a general defense mechanism to prevent the spread of infectious entities such as bacteria or viruses (Lam, 2004). Tomato leaf curl New Delhi Virus (ToLCNDV) resistance protein SlRPT4, a 26S proteasome, is also shown to regulate programmed cell death and ROS production. This is in addition to its inhibitory binding of the viral genome (Sahu et al., 2016).

SnRK1 is a major regulator of energy and nutrients in the plant cell. It is also emerging with a role in plant response to biotic stress (Hulsmans et al., 2016). When challenged with geminivirus infection, *A. thaliana* SnRK1 has been shown to phosphorylate TrAP of CbLCV, delaying and attenuating symptoms (Shen et al., 2014). TrAP is an RNA silencing suppressor and acts as a transcription factor for two other geminiviral proteins (Fondong, 2013). In tomato, the analogue SlSnRK1 was shown to interact with the geminivirus satellite pathogenicity factor βC1. SlSnRK1 is shown to phosphorylate βC1, leading to symptom delay and viral DNA load reduction (Shen et al., 2011). Additionally, SnRK1 phosphorylates the Tomato golden mosaic virus Rep protein and interferes with its dsDNA binding function. This reduces viral DNA replication and symptoms (Shen et al., 2018). These features make SnRK1 an important factor in geminivirus defense. Other aspects of the host response like SUMOylation, senescence response and autophagy (reviewed by Kumar, 2019) may be used against the virus but viable resistance strategies have yet to be developed.

Another receptor kinase that is known to counteract geminivirus infection is the NSP-interacting kinase (NIK) family (Santos et al., 2010). *A. thaliana* NIKs are activated *via* oligomerization and autophosphorylation upon begomovirus infection (Brustolini et al., 2015). The downstream target of NIK is the ribosomal protein L10, which enters the nucleus and downregulates translation-related genes to slow down the infection (Santos et al., 2010; Brustolini et al., 2015). The begomovirus has evolved alongside NIK to suppress its antiviral activity by binding to the critical threonine 474, preventing the phosphorylation required for NIK activation (Santos et al., 2010). This same T474 when changed to phosphomimetic aspartate constitutively activates antiviral response genes, circumvents interference by NSP, and confers tolerance to begomovirus infection (Brustolini et al., 2015). Furthermore, a GTPase that interacts with NSP can also be conceived as a target to develop resistance against geminiviruses (Martins et al., 2020). These responses exemplify that understanding and changing genetic interactions can provide new solutions for disease management.

The status of the plant hormones in response to geminiviruses has not been exhaustively assessed. Hormone changes related to pathogen response fall within the salicylic acid (SA), jasmonic acid (JA), and ethylene pathways (ET), where SA pathway is upregulated during CbLCV infection in *A. thaliana*, JA is downregulated, and ET has both responses in a transcriptomic study (Ascencio-Ibáñez et al., 2008). Plants overexpressing SA (cpr1 mutants) displayed a substantial delay in symptom appearance upon infection, suggesting that plants with SA pathway always on may have partial protection against geminiviruses. On the other side, the CbLCV depletes the production of jasmonate. JA is known for deterring insects and reducing the transmissibility of geminiviruses (Escobar-Bravo et al., 2016) so it could be used to reduce infection and impact transmission of the virus (Sun et al., 2017). The issue is that SA and JA seemed to be antagonistic on the plants, which will make a plant producing both simultaneously at high levels not a viable alternative. Auxins, gibberellins, cytokinins, brassinosteroids, abscisic acid, and strigolactones are all involved in the plant responses. However, not enough information is available yet to derive a putative resistance path.

## Engineered Resistance

Natural defense mechanisms are often utilized to engineer a plant with geminivirus resistance. This review will briefly cover selected examples of engineered geminivirus resistance that interfere with the viral replication cycle. **Figure 1** puts these mechanisms in context with geminiviral life cycle. For a more detailed look at these approaches, see Loriato et al. (2020).

### Interfering With Viral Proteins

Engineered resistance can act on proteins required for viral replication or on viral DNA itself. In an approach known as “immunomodulation”, transgenic plants express antibodies against viral proteins. Single-chain antibodies generated against the geminiviral coat protein were shown to bind and provide resistance *in vivo* (Zakri et al., 2012). Similarly, anti-Rep antibody expression can provide resistance, but the response varies between lines due to variable transgene expression (Safarnejad et al., 2009). DNase 3D8 is a recombinant antibody with single- and double-stranded non-specific DNase activity that has been tested against geminiviruses BCTV and Beet severe curly top virus. Though the expression levels had to be kept low to protect host nucleic acid, 3D8 expression prevented high levels of viral DNA accumulation (Lee et al., 2013).

Peptide aptamers are engineered peptide sequences to be expressed by the host to interfere with the activity of a protein of interest. These are significantly smaller than single-chain antibodies, but work similarly. They have been developed and tested to disrupt functions of geminivirus proteins, one of which is Rep from Tomato Golden Mosaic Virus. The aptamers bound Rep *in vivo* and lowered viral DNA production (Lopez-Ochoa et al., 2006). In transgenic tomato, peptide aptamers were also effective against TYLCV and ToMoV by reducing viral symptoms and viral load (Reyes et al., 2013). Another approach for replication interference is by competition, as has been shown with subgenomic DNA. However, this approach is highly virus-specific, making its practical use limited and inefficient (Stanley et al., 1990; Stenger, 1994).

Viral infection can also be impeded by sequestration of protein targets. Rep can be out-competed for origin binding by artificial zinc-finger proteins with specific DNA binding, and one has been shown with greater affinity for the TYLCV origin than Rep (Mori et al., 2013). A plant expressing it or a similar protein may inhibit geminivirus replication. Mendoza-Figueroa et al. found a globulin-derived peptide with high affinity for the TYLCV origin and that reduced viral load when applied to infected plants. This indicates that the peptide may not need to be expressed by the plant, but rather can be applied exogenously to interfere with Rep activity (Mendoza-Figueroa et al., 2018).

### Viral Responses Against Gene Silencing Rendered by the Host

Gene silencing regulates host processes by eliminating mRNA before translation and is a defense against viral RNA. Dicer-like proteins cleave dsRNA sequences into short interfering RNA (siRNA) molecules 21–24 nt in length (Rey and Fondong, 2018). The siRNA is loaded into a complex with Argonaute proteins, which are then directed to complimentary DNA or RNA sequences. This results in several outcomes, including degradation or sequestration of existing RNAs (post-transcriptional gene silencing) or targeting DNA for methylation (transcriptional gene silencing) (Mathieu and Bender, 2004; Raja et al., 2010). RDRs spread silencing throughout the plant by multiplying the secondary siRNA signal (Rey and Fondong, 2018).

To counteract silencing, geminiviruses have evolved viral suppressors of RNA silencing which are often pathogenicity factors. For example, V2 of Tomato yellow leaf curl China virus interacts with and sequesters secondary siRNAs, hindering the spread of silencing. V2 of TYLCV suppresses gene silencing by interacting with host proteins suppressor of gene silencing 3 and histone deacetylase 6 (Fondong, 2013; Wieczorek and Obrępalska-Stęplowska, 2015; Li et al., 2017; Wang B. et al., 2018). Another example of RNA silencing suppression is TrAP of Mungbean yellow mosaic Indian virus. It slows siRNA production by blocking RDR6-mediated biogenesis of siRNA, binds to Argonaute 1 to prevent its action, and lowers global DNA methylation levels (Buchmann et al., 2009; Kumar et al., 2015). C4 protein of Cotton leaf curl Multan virus, Cassava mosaic viruses, Tomato leaf curl New Delhi virus, and BCTV is also known to suppress silencing (Vanitharani et al., 2004; Ismayil et al., 2018; Vinutha et al., 2018) and can be a symptom determinant in infection (Mills-Lujan and Deom, 2010).

Upon geminivirus challenge, an increase in siRNA is correlated with a decrease in symptoms (Chellappan et al., 2004). Resistance and recovery phenotypes are strongly associated with RNA silencing. The intergenic region of the BCTV is targeted by transcriptional gene silencing, evidenced by a greater proportion of siRNAs produced for this region along with a heavier methylation load in recovered plants (Yadav and Chattopadhyay, 2011; Coursey et al., 2018). There is a significant inverse relation between the viral DNA methylation and the disease progression, measured by symptom severity and viral titer (Rodríguez-Negrete et al., 2009; Yadav and Chattopadhyay, 2011).

Transgenic approaches have been developed in multiple systems to generate plants that produce antiviral siRNAs, artificial miRNAs, long non-coding RNAs, synthetic trans-acting small interfering RNAs, etc., with different levels of success (some reviewed in Kumar and Khan, 2019). This concept has been proven in transgenic beans against Bean golden mosaic virus (Aragão et al., 2013). In general, a viral gene is expressed in the host to initiate PTGS quicker and to a higher degree when challenged by the virus. The plants expressing the gene in hairpin form had the highest levels of resistance and siRNA, with some plants showing no symptoms at all (Leibman et al., 2015; Tomar et al., 2018). However, this is apparently dosage-dependent, as multiple studies have shown that resistance is inversely correlated with siRNA level and can be broken by high viral titer (Vanderschuren et al., 2009; Leibman et al., 2015). Furthermore, it has been described that the resistance is sequence dependent and only covers a single species of virus (Fuentes et al., 2016). This limits the use of the technology but does not preclude its application when the prevalent virus is a single species.

The recent advent of the clustered regularly interspaced short palindromic repeats (CRISPR)/Cas system has introduced new tools for generating resistance to geminiviruses. It has potential as a specific editing tool, and has shown to be effective against several different geminiviruses when expressed in plants (reviewed by Zaidi et al., 2016). In all cases, the presence of the Cas endonuclease and targeted short guide RNAs (sgRNAs) reduced viral titer and symptom development. Studies suggest that the level of Cas9 expression was a deciding factor in the level of symptom reduction (Baltes et al., 2015; Ji et al., 2015). Not only is this system effective in reducing geminivirus infection but confers multiple-virus resistance when the conserved nonanucleotide sequence was targeted. This was demonstrated in a dual infection with TYLCV and BCTV. (Ali et al., 2015).

CRISPR/Cas9 offers a great tool to integrate geminivirus resistance into susceptible plants, yet it is not perfect. Zhang et al. described in 2018 that their high-specificity gRNA was still showing off-target effects when expressed in *A. thaliana*. These were reduced by using a modified gRNA scaffold (expressing the gRNA adjacent to tRNA9met) and using the SpCas9 mutant (Zhang et al., 2018). Another unintended consequence of CRISPR-based viral resistance is the evolution of the virus to evade sgRNA. This adaptation renders the resistance useless (Mehta et al., 2019).

## Conclusions

Although geminiviruses are very successful at infecting their hosts, plants also evolve to overcome or tolerate infection. Protective measures are introduced by humans as we seek to maintain our crop productivity. It is important to continue innovating to keep pace with the rapid viral evolution. Resistance based on DNA sequence is at a disadvantage as it relies on sequence to remain unchanged. These also tend to be very virus-specific, making it difficult to protect crops against mixed infections that are common. Antibody-based systems and peptide aptamers may hold better long-term resistance since they put more indirect evolutionary pressure on the geminivirus genome. Additionally, the peptide aptamers showed a broader base of effectiveness (Reyes et al., 2013), which may be effective at fighting mixed infections. Aptamers can be improved and mixed or re-designed using in silico approaches to increase its affinity. However, the long history of virus/host competitive evolution shows that protein-based resistance can still be overcome. To continue developing more resistant crops, we must gain a greater basic understanding of how these viruses infect the plants and how the plant responds to and harbors viral replication. An ideal resistance would prevent initial viral replication, eliminating the opportunity for the virus to evolve. Furthermore, a combinatorial or additive approach targeting both the virus and the vector may provide with a better opportunity to impair the infection and provide a longer lasting protection to crops.

## Author Contributions

JA-I conceived and supervised the review topics. KB wrote the first draft. All authors contributed to the article and approved the submitted version.

## Funding

This work was partially funded by the Bill and Melinda Gates Foundation (CPT005698) and by the T&E Biochemistry Foundation.

## Conflict of Interest

The authors declare that the research was conducted in the absence of any commercial or financial relationships that could be construed as a potential conflict of interest.
